# Dosimetric comparison of hippocampal-sparing technologies in patients with low-grade glioma

**DOI:** 10.1093/noajnl/vdae131

**Published:** 2024-08-06

**Authors:** Aoife Williamson, Peter Houston, Jennifer Paterson, Anthony J Chalmers, Philip McLoone, Natasha Fullerton, Sin Yee Foo, Allan James, Stefan Nowicki

**Affiliations:** Department of Clinical Oncology, Beatson West of Scotland Cancer Centre, Glasgow, UK; Department of Clinical Oncology, Beatson West of Scotland Cancer Centre, Glasgow, UK; Department of Clinical Oncology, Beatson West of Scotland Cancer Centre, Glasgow, UK; School of Cancer Sciences, University of Glasgow, Glasgow, UK; School of Health & Wellbeing, University of Glasgow, Glasgow, UK; Department of Neuroradiology, Institute of Neurosciences, QEUH, Glasgow, UK; Department of Neuroradiology, Institute of Neurosciences, QEUH, Glasgow, UK; Department of Clinical Oncology, Beatson West of Scotland Cancer Centre, Glasgow, UK; Department of Clinical Oncology, Beatson West of Scotland Cancer Centre, Glasgow, UK

**Keywords:** hippocampal sparing, hyperarc, low-grade glioma, multi-criteria optimizer, photon planning

## Abstract

**Background:**

Radiotherapy (RT) plays an integral role in the management of low-grade gliomas (LGG). Late toxicity from RT can cause progressive neurocognitive dysfunction. Radiation-induced damage to the hippocampus (HCP) plays a considerable role in memory decline. Advancements in photon planning software have resulted in the development of multi-criteria optimization (MCO) and HyperArc technologies which may improve HCP sparing while maintaining planning target volume (PTV) target coverage.

**Methods:**

Three planning methods for hippocampal sparing (HS) were compared, volumetric modulated arc therapy (VMAT) without HS (VMAT_noHS), VMAT with HS (VMAT_HS), MCO with HS (MCO_HS), and HyperArc with HS (HyperArc_HS).

**Results:**

Twenty-five patients were identified. The contralateral HCP was spared in 16 patients and bilateral HCP in 9 patients with superiorly located tumors. All 3 HS planning techniques showed significant reductions in dose to the spared HCP in contralateral cases but only VMAT_HS and MCO_HS achieved this in bilateral cases (*P* < .008). Only MCO_HS was superior to VMAT_HS in lowering the dose to both contralateral HCP and bilateral HCP in all measured metrics (*P* < .008). PTV and OAR (organ at risk) dose constraints were achieved for all plans.

**Conclusions:**

This retrospective dosimetric study demonstrated the feasibility of HS for low-grade glioma. All 3 HS planning techniques achieved significant dose reductions to the spared contralateral hippocampus, but only MCO_HS and VMAT_HS achieved this in bilateral cases. MCO was superior to other planning techniques for sparing both bilateral and contralateral hippocampi.

**Key Points**

- Innovations in photon planning software, with the development of volumetric modulated arc therapy (VMAT), multi-criteria optimization (MCO), and hyperArc technologies, improve HCP sparing for low-grade glioma patients while maintaining PTV target coverage.

**Importance of this Study**

Sparing of the hippocampus (HCP) for low-grade glioma patients undergoing fractionated radiotherapy, presents technical challenges with respect to hippocampal contouring and radiotherapy treatment planning. Innovations in photon planning software, with the development of volumetric modulated arc therapy (VMAT), multi-criteria optimization (MCO), and HyperArc technologies, may improve HCP sparing while maintaining PTV target coverage. Our retrospective dosimetric study demonstrates the feasibility of HCP sparing for low-grade glioma using different photon planning techniques without compromising PTV or other OAR’s. The 3 planning solutions included in our study are available within the majority of radiotherapy facilities. We believe that this paper will be of interest to radiotherapy physicists and radiation oncologists who wish to develop HCP sparing within their departments. This paper highlights the use of this technology, the planning methods involved and identifies the optimal photon planning method for HCP sparing.

Radiation therapy (RT) is integral in the management of primary brain tumors. Low-grade gliomas (LGG) are rare primary brain tumors with relatively slow but infiltrative growth.^1^ The optimum management for LGG patients comprises surgery followed by sequential radiotherapy and chemotherapy which often result in life expectancies of a decade or more.^2,3^ With such a prolonged prognosis the adverse late effects of RT on quality of life and neurocognitive functioning are of crucial importance.

The hippocampi are cerebral structures that are now understood to play a crucial role in cognition.^4,5^ Traditionally, the hippocampus has not been considered an organ at risk (OAR) in radiotherapy planning, unlike other structures such as the optic chiasm or brainstem. However, there is increasing evidence to suggest that irradiation of the hippocampus can result in neurocognitive decline.^6–8^ Studies have identified a relationship between RT dose to the hippocampus, hippocampus volume, and neurocognitive deficit.^9–12^ Preclinical and clinical research suggests that hippocampal sparing (HS) may provide a useful intervention for reducing the adverse cognitive effects of cranial irradiation.^6^ Doses in excess of 7.3 equivalent doses in 2Gy per fraction (EQD2 Gy) to 40% of the bilateral hippocampus have been found to significantly correlate with a decrease in verbal memory performance.^13^

Modern radiotherapy techniques, such as volumetric modulated arc therapy (VMAT), allow improved conformality of the RT dose to the target volume with a reduction of dose to OARs. Recent advancements in photon planning software include multi-criteria optimization (MCO; Varian Medical Systems), an inverse planning technology; and HyperArc (Varian Medical Systems), eclipse treatment planning software feature. Studies have shown that MCO and Hyperarc improve dosimetric quality and efficiency for photon beam planning and delivery for brain metastases, head and neck, prostate, and lung cancers.^14–22^ MCO planning aims to achieve the optimal plan by allowing a planner to trade-off different dose objectives in real-time to reach the best compromise. HyperArc, primarily designed to treat brain metastases, incorporates several specialized functions for radiotherapy planning including automated settings for locating the isocenter, noncoplanar beam arrangements, and optimization of steep dose gradients outside the PTV. HyperArc plans offer the possibility of delivering a more conformal dose to the target while reducing doses to surrounding tissues and have been explored in the context of HS for whole brain irradiation.^23,24^

This dosimetric study was conducted to identify the optimal photon planning method for HS by comparing VMAT, MCO, and Hyperarc plans in patients with LGG.

## Methods

### Study Design

This is a retrospective planning study comparing 3 different HS planning techniques in patients with low-grade glioma. Patients were planned using the following techniques: to provide a contemporary comparator, original plans (VMAT_noHS) were recalculated using an updated dose calculation algorithm subsequently adopted by the department (Acuros 15.5.07). Three planning methods for HS were compared, VMAT without HS (VMAT_noHS):VMAT with HS (VMAT_HS), MCO with HS (MCO_HS); and HyperArc with HS (HyperArc_HS). Each HS technique was then compared to the recalculated original plan. For patients in whom the planning target volume (PTV) overlapped the hippocampus, we sought to spare only the hippocampus on the contralateral side. In other patients with PTV’s not including one or both hippocampus, bilateral HS was the objective.

### Patient Selection and Methods

For this study, all adult patients with histopathologically confirmed WHO grade 2 oligodendroglioma or grade 2 astrocytoma who were treated with radiotherapy in our local center between 2015 and 2019 were included in this study. All patients were identified retrospectively and all patients who fitted this criteria were included.

The patient characteristics are shown in Table 1.

**Table 1.** Patient Characteristics

Patient characteristics (*n* = 25)		
Characteristics	Number (%) or median (range)	
Age (years)	39	(27–55)
*Sex*		
Male	9	(36.0)
Female	16	(64.0)
PTV (cm^3^)	277.4	(174.0-555.4)
Hippocampal volume cm^3^	1.58	(1.12-2.62)
*Tumor localization*		
Right	12	(48.0)
Left	13	(52.0)
*Tumor location*		
Frontal	9	(36.0)
Temporal	7	(28.0)
Fronto-temporal	6	(24.0)
Parietal	1	(4.0)
Temporal-parietal	1	(4.0)
Parietal-occipital	1	(4.0)
*Pathology*		
Oligodendroglioma	14	(56.0)
Astrocytoma	11	(44.0)
*Surgery*		
Debulking	18	(72.0)
Partial debulk	7	(28.0)

### Patient Simulation and Outlining

All patients were immobilized in a supine position using a 3-point thermoplastic fixation device (Civco Medical Solutions). Patients underwent a 1.5 mm slice non-contrast computed tomography (CT) scan. All patients also underwent imaging on a 1.5 tesla MR scanner (Philips Medical Systems, Best), with 1.5 mm axial T2-weighted and gadolinium contrast-enhanced T1-weighted sequences acquired. CT simulation and MR images were co-registered on Varian Aria V15.1 using a rigid registration algorithm. The gross tumor volume was defined as the area of increased signal intensity on T2 MRI imaging plus the surgical resection cavity. A margin of 15 mm was added to define the clinical target volume (CTV). The CTV margin was applied in all directions of likely tumor spread along the white matter tracts but was modified to respect anatomical barriers. The PTV was generated by adding a geometric isotropic margin of 3 mm. All contours were delineated by an experienced neuro-oncology clinical oncologist and each delineation was peer-reviewed by the local neuro-oncology team.

The hippocampus was retrospectively delineated by a neuro-radiologist on gadolinium contrast-enhanced T1 weighted MRI. Delineation was performed on the axial slices as per the RTOG 0933 atlas.^25^ The contours were then reviewed and verified in sagittal, coronal and axial planes by another neuro-radiologist. A hippocampal avoidance zone was generated by adding a 3 mm isotropic margin.

### Treatment Planning

#### Original clinical VMAT plans without hippocampus optimization.—

The clinically delivered VMAT plans (VMAT_noHS) were created by experienced planners between 2015 and 2019 using Eclipse Treatment Planning System (TPS; version 13.6 – 15.5 Varian Medical Systems Palo Alto). Plans consisted of 2 full coplanar arcs with a 30° complimentary collimator tilt; the hippocampus was not considered in plan optimization. To reduce inter-operator and TPS version variability, for this study the original plans were re planned by 2 experienced planners (>10 years of experience) using our departments RapidPlan Model (RP; version 15.5.11) and calculated with Acuros 15.5.07. The same 2 planners performed all subsequent planning and analysis. The field geometry of the plans was unchanged.

#### VMAT plans with hippocampus optimization.

—The VMAT_noHS plans described above were then created in Eclipse 15.5, using the RP model to generate PTV and OAR objectives. HS was then introduced by manually adding hippocampus optimization objectives. Planners aimed to achieve hippocampus D40% < 12 Gy without compromising PTV coverage or other OAR constraints. The D40% value of <12 Gy was selected as this was the lowest mean dose to the hippocampus achieved in published peer-reviewed papers pertaining specifically to HS for low and high-grade gliomas.^26,27^ After careful consideration and discussion within our clinical team, it was decided to proceed with the D40% value of <12 Gy in order to robustly challenge the planning systems to investigate the feasibility of this constraint.

VMAT plans with hippocampus sparing (VMAT_HS) consisted of 2 full arcs with a 30° complementary collimator tilt.

#### MCO planning process.—

MCO planning enables optimization of treatment plans by allowing the planner to explore the Pareto surface of various dose objectives. The Pareto surface is explored using a slider bar, with visual dose volume histogram (DVH) information and selected dose objectives displayed to guide the selection of an optimal solution. With the VMAT_HS plan acting as a starting point for the MCO process, highest priority was given to not compromising PTV coverage or OAR constraints and then minimizing hippocampus dose, primarily D40%, to generate the MCO_HS plan retaining a 2 arc coplanar geometry. Within these criteria, the final determination of the optimal solution was left to the planners judgment.

#### HyperArc planning.

—HyperArc is an Eclipse TPS feature which is primarily designed to treat brain metastases in a highly conformal, efficient, and automated manner. HyperArc beam arrangements are noncoplanar and designed to be collision risk-free due to the use of a Q-Fix Encompass immobilization device which is modeled in the Eclipse TPS. As these retrospective patients were scanned prior to the use of the Encompass immobilization system, an Encompass structure set was inserted into the dataset to allow the use of HyperArc planning. HyperArc_HS plans were created using the HyperArc (v15) plan creation wizard to place the isocentre of the noncoplanar beams in a collision-free zone. Beam arrangements varied based on the geometry of the PTV but typically 4–5 noncoplanar half arcs were used with the collimator angles optimized for MLC coverage of the target. Rapid Plan (RP) was used to generate PTV and OAR optimization objectives while planners manually attempted to spare hippocampus dose by prioritizing D40% during optimization. For all plans, final dose distributions were calculated with Acuros 15.5.07 at a 2.5 mm grid size and were optimized and calculated on a TrueBeam STX at 6MV with HD-MLC. The TrueBeam STX was chosen because it is HyperArc enabled and would allow clinical delivery of all calculated plans for the purposes of quality assurance.

#### Dosimetric and plan quality comparison analysis.

—For all patients, the prescription dose to the PTV was 50.4 Gy in 28 fractions. All plans were normalized to a median PTV dose of 100%. PTV and OAR dose constraints are detailed in Appendix 1. The PTV was given priority over OARs and PTV coverage was not compromised in any of the plans. With regards to HS, the following metrics were assessed: mean dose (Dmean), point max of the hippocampus (Dmax), and D40%. In cases where the ipsilateral hippocampus was within the PTV or adjacent to the PTV, the dosimetric criteria for the contralateral hippocampus were prioritized. The brain stem, optic chiasm, and optic apparatus were also delineated and these established organs at risk were prioritized over the hippocampal dose constraint. General plan quality was assessed by experienced operators. DVH data for all evaluated parameters were recorded and analyzed.

### Statistical Analysis

Each of the PTV targets (D2, D5, D95, D99, and mean dose), hippocampus (D10, D20, D30, D40, mean and maximum dose), and other OAR indices were summarized using medians and interquartile ranges (IQR). Differences in patient index values between all planning techniques were assessed using the Friedman test (which is a nonparametric repeated measures ANOVA). A statistical significance level of 0.05 was used for this test. Differences between pairs of planning techniques were assessed using the Wilcoxon signed rank test. For these multiple pairwise comparisons, a Bonferroni-adjusted statistical significance level of 0.008 was used.

### Ethics Approval and Consent to Participate

This study was a retrospective radiotherapy planning study using anonymized patient data. Ethical approval was not required as per Beatson West of Scotland Cancer Center Research & Development guidelines. This research was conducted ethically in accordance with the World Medical Association Declaration of Helsinki. This work did not require written patient consent.

## Results

Of the 25 patients included, bilateral HS was deemed appropriate in 9. For the other 16 patients, only the contralateral hippocampus was contoured due to the overlap of the ipsilateral hippocampus and the PTV. Dose statistics are quoted for contoured hippocampus volumes. In Table 2, the PTV target indices are shown for all planning techniques. These were achieved for all plans included in the study. No clinically significant differences in median values of PTV dose measures were observed between different planning techniques. For all 25 patients, there was no statistically significant difference between planning techniques in terms of the monitor units delivered.

**Table 2.** Median (IQR) D2, D5, D95, D99, Mean Dose and MU for Planning Target Volume

	VMAT_noHS median (IQR)	VMAT_HS Median (IQR)	HyperArc_HS median (IQR)	MCO_ HS median (IQR)	Friedman test
D2 < 107%	102.2 (101.5–103.0)^3^	102.3 (101.6–102.9)^3^	102.7 (102.0–103.2)^1^^,^^2^^,^^4^	102.3 (101.7–102.7)^3^	*P* = .0001
D5 < 105%	101.4 (101.1–101.9)^3^	101.6 (101.2–102.0)^3^	101.7 (101.4–102.1)^1^^,^^2^	101.4 (101.3–101.9)	*P* = .0002
D95 > 95%	97.9 (97.4–98.2)	97.7 (97.4–98.2)	97.7 (96.9–98.0)^4^	98.2 (97.9–98.3)^3^	*P* = .037
D99 > 90%	96.0 (95.1–96.8)^4^	95.7 (94.9–96.4)^4^	95.5 (94.1–96.1)^4^	96.7 (96.2–97.0)^1^^,^^2^^,^^3^	*P* = .0038
Mean%	99.9 (99.9–99.9)	99.9 (99.9–99.9)	99.9 (99.8–100)	99.9 (99.9–100)	*P* = 0.133
MU	378 (341–395)	382 (352–407)	364 (338–401)	394 (361–412)	*P* = 0.106

Pairwise comparisons Wilcoxon sign rank test—*P* < .008 compared to

^1^VMAT_noHS,

^2^VMAT_HS,

^3^MCO_HS,

^4^HyperArc_HS.

HS, hippocampal sparing; MCO, multi-criteria optimization; VMAT, volumetric modulated arc therapy.

### Dose to Bilateral Hippocampus—9 Cases

**Figure 1.**
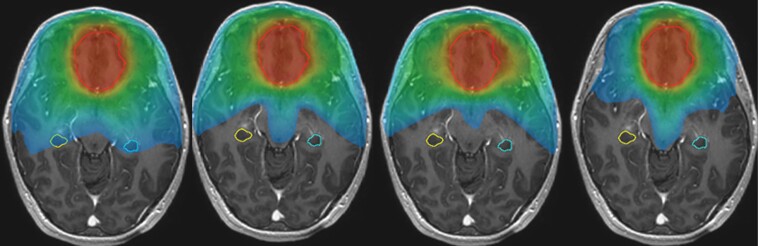
BiLateral hippocampal sparing (images V12 Gy color wash). From left to right: VMAT_noHS mean dose 12.7 Gy, VMAT_HS mean dose 5.1 Gy, MCO_HS mean dose 3.3 Gy, HyperArc _HS mean dose 4.3 Gy.

The calculated parameters for the bilateral hippocampus are shown in Table 3. Left and right hippocampal volumes were considered as a single overall structure. All 3 HS planning techniques showed dose reductions compared to VMAT_noHS, but only MCO_HS achieved a statistically significant difference (*P* < .008) across all measured parameters (Figure 1). There were statistically significant differences between HS techniques VMAT_HS and MCO_HS, but no statistically significant difference between these techniques and HyperArc_HS, even though HyperArc_HS tended to achieve the lowest mean dose. When compared to VMAT_noHS, there was a median decrease in D40% of 5 Gy (IQR 0.5–10.4) for VMAT_HS, 7.4 Gy (IQR 1.5–13.7) for MCO_HS, and 6.9 Gy (IQR −1.1–11.3) for HyperArc_HS. The median mean dose to the bilateral hippocampi with VMAT_noHS was 12.1 Gy (IQR 5.0–20.5). This value was reduced by 4.6 Gy (IQR 2.3–12.4), 6.1 Gy (IQR 1.9–10.6) and 6.5 Gy (IQR −0.4–12.9) by VMAT_HS, MCO_HS, and HyperARC_HS, respectively. HyperArc_HS showed the lowest median maximum dose to bilateral hippocampi of 9.1 Gy (IQR 7.8–16.8). Considering the HS techniques used for these 9 cases, the target D40% of <12 Gy was achieved by 8 VMAT_HS, 8 MCO_HS, and 8 HyperArc_HS plans (Appendix 2), whereas only 3 of the 9 VMAT_noHS plans achieved the target D40% constraint.

**Table 3.** Bilateral Hippocampus Median (IQR) Mean Dose, D10, D20, D30, D40, and Maximum Dose

	VMAT_noHS	VMAT_HS	MCO_HS	HyperArc_HS
mean	12.1 (5.0–20.5)^2^^,^^3^	7.5 (3.1–7.9)^1^^,^^3^	6.0 (2.7–7.3)^1^^,^^2^	5.6 (5.3–8.0)
D10	14.9 (10.0–25.7)^2^^,^^3^^,^^4^	8.4 (4.3–10.2)^1^^,^^3^	8.2 (3.6–9.1)^1^^,^^2^	7.0 (6.3–10.0)^1^
D20	14.1 (7.6–23.8)^2^^,^^3^	8.2 (3.8–9.1)^1^^,^^3^	7.7 (3.2–8.4)^1^^,^^2^	6.4 (6.1–9.1)
D30	13.4 (5.6–22.4)^2^^,^^3^	8.0 (3.5–7.7)^1^^,^^3^	6.8 (3.0–7.7)^1^^,^^2^	6.0 (5.8–8.5)
D40	12.7 (4.3–21.3)^3^	7.7 (3.2-8.1)^3^	5.3 (2.8-7.5)^1^^,^^2^	5.8 (5.5–7.7)
Max	22.4 (16.8–32.6)^2^^,^^3^^,^^4^	9.8 (7.3–16.5)^1^^,^^3^	9.8 (5.5–14.2)^1^^,^^2^	9.1 (7.8–16.8)^1^

Pairwise comparisons Wilcoxon sign rank test—*P* < .008 compared to

^1^VMAT_noHS,

^2^VMAT_HS,

^3^MCO_HS

^4^HyperArc_HS.

HS, hippocampal sparing; MCO, multi-criteria optimization; VMAT, volumetric modulated arc therapy.

### Dose to Contralateral Hippocampus—16 Cases

**Figure 2.**
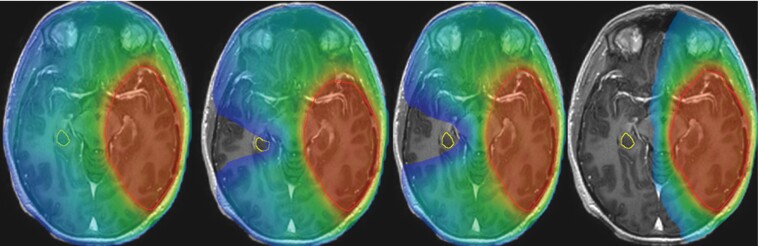
Contralateral hippocampal sparing (images V12 Gy color wash). From left to right: VMAT_noHS mean dose 20.5 Gy, VMAT_HS mean dose 7.0 Gy, MCO_HS mean dose 4.5 Gy, HyperArc _HS mean dose 5.5 Gy.

Table 4 shows all the calculated parameters for the contralateral hippocampus. All 3 HS planning techniques showed significant dose reductions to the spared contralateral hippocampus (Figure 2). MCO_HS achieved the lowest median hippocampal mean dose and median D40%. The median mean dose was 23.2 Gy (IQR 19.0–29.3) with VMAT_noHS; this was reduced to 10.2 Gy (IQR 8.6–12.4) by VMAT_HS, 9.1 Gy (IQR 6.6–11.5) by MCO_HS and 10.0 Gy (IQR 5.0–11.3) by HyperArc_HS. The median D40% dose of 23.3 Gy (IQR 20.0–28.8) achieved by VMAT_noHS was reduced to 9.8 Gy (IQR 7.8–11.1) by VMAT_HS, 7.8 Gy (IQR 6.6–11.5) by MCO_HS and 8.9 Gy (IQR 5.0–11.3) by HyperArc_HS. The median reduction in D40% dose was greatest with MCO_HS (15.5 Gy) compared to median reductions of 13.5 and 14.4 Gy with VMAT_HS and HyperArc_HS, respectively. HyperArc_HS achieved the lowest median maximum dose to the contralateral hippocampus (18.4G y—IQR 7.0–42.7). Amongst the 16 contralateral hippocampal cases, only one of the VMAT_noHS plans met the D40% <12 Gy constraint. Amongst the HS techniques, this number increased to 13 for VMAT_HS, 14 for MCO_HS, and 15 for Hyperarc_HS (Appendix 2).

**Table 4.** Contralateral Hippocampus Median (IQR) Mean Dose, D10, D20, D30, D40, and Max Dose

	VMAT_noHS	VMAT_HS	MCO_HS	HyperArc_HS
mean	23.2 (19.0–29.3)^2^^,^^3^^,^^4^	10.2 (8.6–12.4)^1^^,^^3^	9.1 (6.6–11.5)^1^^,^^2^	10.0 (5.0–11.3)^1^
D10	28.3 (22.8–37.9)^2^^,^^3^^,^^4^	14.2 (10.3–24.2)^1^^,^^3^	12.9 (8.0–20.1)^1^^,^^2^	11.8 (5.6–20.8)^1^
D20	24.8 (21.9–32.4)^2^^,^^3^^,^^4^	12.3 (9.5–17.2)^1^^,^^3^	10.6 (7.5–15.6)^1^^,^^2^	10.8 (5.4–13.8)^1^
D30	24.0 (20.5–30.7)^2^^,^^3^^,^^4^	10.9 (9.1–12.6)^1^^,^^3^	9.0 (7.0–11.5)^1^^,^^2^	9.5 (5.3–11.9)^1^
D40	23.3 (20.0–28.8)^2^^,^^3^^,^^4^	9.8 (7.8–11.1)^1^^,^^3^	7.8 (6.4–10.6)^1^^,^^2^	8.9 (5.2–10.8)^1^
Max	33.8 (26.9–47.1)^2^^,^^3^^,^^4^	24.9 (13.2–41.1)^1^^,^^3^	20.7 (7.0–42.7)^1^^,^^2^	18.4 (7.0–42.7)^1^

Pairwise comparisons Wilcoxon sign rank test—*P* < .008 compared to

^1^VMAT_noHS,

^2^VMAT_HS,

^3^MCO_HS

^4^HyperArc_HS.

HS, hippocampal sparing; MCO, multi-criteria optimization; VMAT, volumetric modulated arc therapy.

### Dose to Normal Brain and OARs

The difference between the brain (-PTV) doses for the 4 techniques was small but statistically significant (*P* < .0001; Appendix 3 and 4). All planning techniques achieved dose constraints for orbit, optic nerve, and brainstem but not the lens. MCO_HS delivered the lowest median doses to the brainstem, optic chiasm, and lens. For contralateral sparing plans, the differences between the median brain (-PTV) dose for the 4 techniques were small and nonsignificant. All other OAR dose constraints were achieved by all 4 planning techniques.

### Correlation Between PTV Size and Dose to Hippocampus

Spearman correlation coefficients for the association between PTV size and dose to hippocampus are shown in Appendix 5. For bilateral cases, no significant correlation between the size of the PTV and the hippocampal dose (mean and D40) was observed in any of the planning techniques. For contralateral cases, statistically significant strong correlations (*P* < .01) were found between PTV size and hippocampal D40 for VMAT_HS, MCO_HS, and HyperArc_HS (rho = 0.724, 0.638, 0.698, respectively). However, no statistically significant correlation was observed with the contralateral mean dose.

### Influence of PTV Localization on Hippocampal Dose

No significant influence of tumor localization was observed for hippocampal mean and D40 doses for both unilateral and bilateral cases. For temporal lesions, the HS techniques were associated with higher D40 doses compared to lesions without temporal involvement but this was not statistically significant (Appendix 6).

## Discussion

The purpose of this dosimetric study was to investigate the feasibility of HS for LGG patients and to compare the ability of different photon planning methods to achieve it. We found that VMAT plans with HS could significantly reduce the dose to either contralateral or bilateral hippocampi, the latter being dependent on the proximity of the ipsilateral hippocampus to the PTV, while maintaining PTV coverage and other OAR constraints. No attempt was made to preserve the ipsilateral hippocampus when within or in close proximity to the PTV given the possibility of infiltration by the tumor (16 cases). For the 9 cases where both delineated hippocampal structures were distant to the PTV, bilateral sparing was carried out and the average distance from the inferior slice of the PTV to the superior slice of the delineated hippocampal volume was 2.3cm (0.7–3.5cm).

In our study, for both bilateral and contralateral sparing cases, only MCO_HS achieved statistically significant reductions in hippocampal dose parameters compared to the original VMAT and VMAT _HS plans. MCO_HS showed statistically significant dose reductions across all parameters compared to VMAT_noHS for bilateral HS plans. VMAT _HS achieved statistically significant reductions in mean and maximum hippocampal doses compared to the original plan. HyperArc_HS only achieved a statistically significant reduction in maximum hippocampal dose when compared to VMAT_noHS.

Comparing the 3 HS techniques, only MCO_HS showed a consistent statistically significant difference (*P* < .008) across all measured parameters when bilateral HS was attempted. This highlights the potential benefits of the MCO planning process, which allows planners to minimize hippocampal dose, primarily D40%, with 2 arcs coplanar geometry. The range in dose-sparing metrics for the bilateral hippocampi is indicative of the large variations in PTV and hippocampus geometry that are commonly observed. This may also account for the higher dose-sparing metrics achieved by hyperarc when delivering noncoplanar beams.

For contralateral hippocampus sparing, all 3 techniques were statistically superior to the original VMAT_noHS plans. Of the 3 planning methods studied, MCO_HS generally achieved the lowest contralateral hippocampus dose and was the only technique that showed significant reductions compared to VMAT_HS. HyperArc_HS showed consistently lower Dmax for contralateral HS compared to the other 2 techniques. This reflects the benefits of a noncoplanar treatment approach that allows the opportunity to limit lateral entry and exit doses to a greater degree than coplanar planning techniques.

To our knowledge, this study is the first to compare VMAT_HS with multi-criteria optimizer (MCO) and HyperArc planning technology in this patient group. However, previous studies have investigated HS using VMAT and IMRT in low and high-grade glioma. In a dosimetric study evaluating hippocampal-sparing radiotherapy for 18 patients with grades II and III gliomas using IMRT,^28^ a mean hippocampal dose of less than 30 Gy was achieved in 14 out of 18 patients, with a mean dose to contralateral hippocampus of 24.9 Gy. The authors identified that a PTV volume of less than 420.5 cm^3^ was the only predictive factor in achieving HS. In another dosimetric study comparing 3D conformal radiotherapy and VMAT planning in high-grade gliomas,^29^ a 36% reduction to the contralateral HCP was achieved with VMAT (mean dose 14.7 Gy). This study observed that, for VMAT planning, parietal lobe tumor location and larger PTV size (median volume 393.8 cm^3^) were predictors of higher hippocampal dose (*P* < .05). A dosimetric case study of a left frontal grade II astrocytoma showed that VMAT planning with HS (standard coplanar) reduced the mean dose to the contralateral hippocampus from 29.9 to 12.6 Gy.^26^ An IMRT planning study to assess the feasibility of sparing the hippocampus and limbic circuit during partial brain radiotherapy for 5 hemispheric LGG reduced the mean dose from 32.6 to 12 Gy.^27^ Other studies have identified significant correlations between PTV size and mean dose to the contralateral hippocampus.^29^ In our study, a significant dose correlation was observed between PTV size and dose to the contralateral D40% for VMAT_HS, MCO_HS, and HyperArc_HS, but no correlation was found for the contralateral mean dose. No correlation was observed for bilateral plans, suggesting that larger PTV’s do not influence D40% or mean dose when distant (superior) to the hippocampi.

The hippocampus median dose values achieved in our study were lower than those reported in other studies.^26–28^ These studies (previously discussed) included a variety of pathologies, target volumes, doses, and fractionation which may have resulted in higher doses to the hippocampus. Our study involved only grade II gliomas treated with a dose of 50.4 at 1.8 Gy per fraction with the HCP D40% constraint of <12 Gy. The equivalent EQD2 for 1.8 Gy per fraction for the 7.3 Gy threshold identified in the Gondi paper (alpha beta normal brain tissue = 2) was 7.7 Gy. With bilateral HS plans the D40% achieved was 7.7 Gy for VMAT_HS, 5.3 Gy for MCO_HS, and 5.8 Gy for Hyperarc_HS. For contralateral-only plans, MCO_HS achieved median D40% of 7.8 Gy compared with 9.8 Gy and 8.9 Gy for VMAT_HS and HyperArc_HS, respectively. This indicates that it is possible to achieve EQD2 D40% values of 7.7 Gy with all 3 HS techniques when bilateral HS is possible, and with MCO when contralateral HS is attempted.

The major shortcoming of any retrospective planning study is the inherent plan bias. Planner experience is crucial because the plan optimization process is user-dependent. We used various strategies to mitigate this bias: RP model knowledge-based planning was used as a planning aid for HS coplanar techniques, 2 experienced planners were involved, dose objective documentation was created and planners verified that all dose constraints were met. High-quality RP models result in improved plan quality, optimal target coverage, reduced OAR doses, and substantially reduced planning times.^30^ The RP model utilized for this study is built from a dataset of 79 patients, has been internally validated and is routinely used for clinical patients. It is also worth noting that a larger cohort of patients would be beneficial to validate the findings of this study but this is challenging due to the rare nature of this disease. Planning time was not captured for this study. Therefore, we have no insight into the time taken to plan a patient with each of the 4 planning methods. This would be helpful information for radiotherapy departments considering the implementation of HS with limited resources.

The prospective study by Gondi et al,^13^ which included benign and low-grade brain tumors treated with fractionated radiotherapy, observed a relationship between EQD2 to the bilateral hippocampi and likelihood of long-term memory impairment at 18-month follow-up. A normal tissue complication probability (NTCP) of 66.7% was observed when the D40% exceeded an EQD2 of 7.3 Gy. Other studies have attempted to quantify the hippocampal NTCP model within a group of LGG patients. Analysis of data from the EORTC 22033-26033 trial^31^ revealed no difference in incidence of a cognitive event between patients receiving D40% above vs. below the median (47.2 Gy; 14 vs. 25%, *P* = .68).^32^ Median follow-up in the EORTC 22033-26033 trial was 18 months, which may be considered short in the context of late neurocognitive toxicity. Previous studies have identified neurocognitive deficits occurring 5 years or more after fractionated radiotherapy.^33,34^ Further studies are required in low-grade gliomas to elucidate the relationship between the dose received by the hippocampus during photon radiotherapy and neurocognitive dysfunctions. A prospective longitudinal study utilizing HS with MCO and neurocognitive assessment in grade 2 oligodendroglioma and astrocytoma patients is in development within our local center. The authors have identified the benign tumor type meningioma as another disease indication that could benefit from the use of HS. A dosimetric study is planned for this patient group.

## Conclusion

This retrospective dosimetric study performed on 25 patients demonstrates the feasibility of HS for low-grade glioma using different photon planning techniques without compromising PTV or other OAR’s. All 3 HS planning techniques showed significant benefits when contralateral hippocampus sparing was suitable, but only MCO_HS and VMAT_HS showed benefit when bilateral HS was attempted. MCO_HS showed the largest reduction in median hippocampal D40% in both unilateral and bilateral cases, which could potentially lead to a clinically relevant reduction of late neurocognitive side effects, although further research would be required to confirm this.

## Supplementary Material

vdae131_suppl_Supplementary_Appendix_S1

vdae131_suppl_Supplementary_Appendix_S2

vdae131_suppl_Supplementary_Appendix_S3

vdae131_suppl_Supplementary_Appendix_S4

vdae131_suppl_Supplementary_Appendix_S5

vdae131_suppl_Supplementary_Appendix_S6
