# Supplementary material for: Dosimetric comparison of hippocampal-sparing technologies in patients with low-grade glioma
Source: Neurooncol Adv. 2024 Aug 6;6(1):vdae131. doi: 10.1093/noajnl/vdae131 (PMC11364934; doi:10.1093/noajnl/vdae131)
Supplement: vdae131_suppl_Supplementary_Appendix_S1 [file vdae131_suppl_supplementary_appendix_s1.docx]

Appendix 1 PTV & OAR Dose constraints

| **Organ** | **Dose Constraint** |
| --- | --- |
| PTV | D99% ≥ 90% |
| PTV | D95% ≥ 95% |
| Brain | D10% ≤ 57Gy |
| Brain | Mean Dose ≤ 24Gy |
| Brainstem | D5% ≤ 57Gy |
| Brainstem | Mean dose ≤ 52Gy |
| Optic Chiasm | D1% ≤ 54Gy |
| Optic Nerve | D1% ≤ 54Gy |
| Orbit | D1% ≤ 30Gy |
| Lens | D1% ≤ 6Gy |
| Hippocampus | D40% ≤ 12Gy |
