# Supplementary material for: Dosimetric comparison of hippocampal-sparing technologies in patients with low-grade glioma
Source: Neurooncol Adv. 2024 Aug 6;6(1):vdae131. doi: 10.1093/noajnl/vdae131 (PMC11364934; doi:10.1093/noajnl/vdae131)
Supplement: vdae131_suppl_Supplementary_Appendix_S2 [file vdae131_suppl_supplementary_appendix_s2.docx]

Appendix 2. Number of plans (both contralateral and bilateral) which meet target indices and OAR constraints

|  |  |  | Number of plans which meet constraint n (%) | | | | | | | |
| --- | --- | --- | --- | --- | --- | --- | --- | --- | --- | --- |
| Organ | constraint | N | VMAT | | VMAT_HS | | MCO_HS | | HyperArc_HS | |
| Contra Hippo | Mean≤45 | 16 | 16 | (100.0) | 16 | (100.0) | 16 | (100.0) | 16 | (100.0) |
|  | D10≤50 | 16 | 16 | (100.0) | 16 | (100.0) | 16 | (100.0) | 16 | (100.0) |
|  | D20≤40 | 16 | 16 | (100.0) | 16 | (100.0) | 16 | (100.0) | 16 | (100.0) |
|  | D30≤20 | 16 | 3 | (18.8) | 14 | (87.5) | 14 | (87.5) | 15 | (93.8) |
|  | D40≤12 | 16 | 1 | (6.3) | 13 | (81.3) | 14 | (87.5) | 15 | (93.8) |
|  | Max≤50.4 | 16 | 14 | (87.5) | 16 | (100.0) | 16 | (100.0) | 15 | (93.8) |
| BRAIN-PTV | Mean≤24 | 25 | 25 | (100.0) | 25 | (100.0) | 24 | (96.0) | 25 | (100.0) |
|  | D10≤50 | 25 | 25 | (100.0) | 25 | (100.0) | 25 | (100.0) | 25 | (100.0) |
| Brainstem | Mean≤52 | 25 | 25 | (100.0) | 25 | (100.0) | 25 | (100.0) | 25 | (100.0) |
|  | D5≤57 | 25 | 25 | (100.0) | 25 | (100.0) | 25 | (100.0) | 25 | (100.0) |
| Hippo Bilateral | Mean<45 | 9 | 9 | (100.0) | 9 | (100.0) | 9 | (100.0) | 9 | (100.0) |
|  | D10≤50 | 9 | 9 | (100.0) | 9 | (100.0) | 9 | (100.0) | 9 | (100.0) |
|  | D20≤40 | 9 | 8 | (88.9) | 9 | (100.0) | 9 | (100.0) | 9 | (100.0) |
|  | D30≤20 | 9 | 5 | (55.6) | 8 | (88.9) | 8 | (88.9) | 8 | (88.9) |
|  | D40≤12 | 9 | 3 | (33.3) | 8 | (88.9) | 8 | (88.9) | 8 | (88.9) |
|  | Max<50.4 | 9 | 9 | (100.0) | 9 | (100.0) | 9 | (100.0) | 9 | (100.0) |
| PTV | D2≤107 | 25 | 25 | (100.0) | 25 | (100.0) | 25 | (100.0) | 25 | (100.0) |
|  | D5≤105 | 25 | 25 | (100.0) | 25 | (100.0) | 25 | (100.0) | 25 | (100.0) |
|  | D95≥95 | 25 | 25 | (100.0) | 25 | (100.0) | 25 | (100.0) | 25 | (100.0) |
|  | D99≥99 | 25 | 25 | (100.0) | 25 | (100.0) | 25 | (100.0) | 25 | (100.0) |
| Contra lens | D1≤6 | 16 | 13 | (81.3) | 12 | (75.0) | 13 | (81.3) | 15 | (93.8) |
| Lat lens | D1≤6 | 16 | 12 | (75.0) | 10 | (62.5) | 11 | (68.8) | 12 | (75.0) |
| Bilat left lens | D1≤6 | 9 | 8 | (88.9) | 8 | (88.9) | 8 | (88.9) | 9 | (100.0) |
| Bilat right lens | D1≤6 | 9 | 8 | (88.9) | 8 | (88.9) | 8 | (88.9) | 8 | (88.9) |
| Contra ON | D1≤54 | 16 | 16 | (100.0) | 16 | (100.0) | 16 | (100.0) | 16 | (100.0) |
| Lat ON | D1≤54 | 16 | 16 | (100.0) | 16 | (100.0) | 16 | (100.0) | 16 | (100.0) |
| Bilat left ON | D1≤54 | 9 | 9 | (100.0) | 9 | (100.0) | 9 | (100.0) | 9 | (100.0) |
| Bilat right ON | D1≤54 | 9 | 9 | (100.0) | 9 | (100.0) | 9 | (100.0) | 9 | (100.0) |
| Contra ORBIT | D1≤30 | 16 | 16 | (100.0) | 16 | (100.0) | 15 | (93.8) | 16 | (100.0) |
| Lat ORBIT | D1≤30 | 16 | 15 | (93.8) | 14 | (87.5) | 14 | (87.5) | 14 | (87.5) |
| Bilat left ORBIT | D1≤30 | 9 | 9 | (100.0) | 9 | (100.0) | 9 | (100.0) | 9 | (100.0) |
| Bilat right ORBIT | D1≤30 | 9 | 9 | (100.0) | 9 | (100.0) | 9 | (100.0) | 9 | (100.0) |
