# Supplementary material for: Dosimetric comparison of hippocampal-sparing technologies in patients with low-grade glioma
Source: Neurooncol Adv. 2024 Aug 6;6(1):vdae131. doi: 10.1093/noajnl/vdae131 (PMC11364934; doi:10.1093/noajnl/vdae131)
Supplement: vdae131_suppl_Supplementary_Appendix_S3 [file vdae131_suppl_supplementary_appendix_s3.docx]

Appendix 3: Dosimetric comparison of bilateral plans (median & IQR)

| Dosimetric comparison of bilateral plans (median & IQR) | | | | |
| --- | --- | --- | --- | --- |
|  | VMAT | VMAT_HS | MCO_HS | HyperArc |
| Brain -PTV mean dose <57Gy | 16.7 (15.6-18.6) | 16.0 (15.0-17.6) | 15.7 (15.1-17.6) | 14.1 (14.0-14.6) |
| Brain-PTV D10 <24Gy | 35.4 (33.9-37.0) | 35.1 (34.0-36.8) | 36.1 (34.1-37.0) | 31.8 (30.9-32.9) |
| Brainstem mean | 4.9 (1.9-9.9) | 4.6 (1.7-7.4) | 3.4 (1.6-6.9) | 9.1 (5.9-9.7) |
| Brainstem D5 | 14.4 (5.2-25.7) | 13.4 (3.8-19.5) | 9.6 (3.1-17.4) | 16.7 (12.7-17.1) |
| Chiasm D1 | 23.2 (13.2-49.4) | 23.9 (7.9-47.4) | 18.9 (5.2-44.4) | 30.8 (18.7-46.8) |
| Bilat left Lens D1 | 3.3 (1.9-5.3) | 4.1 (1.8-5.5) | 3.2 (1.6-4.7) | 5.1 (5.0-5.3) |
| Bilat RT Lens D1 | 4.4 (1.5-5.4) | 4.8 (1.7-5.8) | 3.2 (1.6-5.0) | 5.2 (5.0-5.6) |
| Bilat left ON D1 | 8.4 (4.6-41.0) | 7.9 (4.4-40.7) | 7.1 (3.8-26.6) | 27.4 (15.8-35.8) |
| Bilat right ON D1 | 8.9 (3.8-50.6) | 9.5 (3.9-49.5) | 10.8 (3.4-46.7) | 22.1 (13.3-48.8) |
| Bilat left Orbit D1 | 9.4 (5.9-26.2) | 9.8 (3.2-27.6) | 11.0 (2.8-22.1) | 10.3 (9.3-15.9) |
| Bilat right Orbit D1 | 16.3 (4.0-25.9) | 16.5 (5.3-26.5) | 13.7 (3.4-26.6) | 9.9 (8.1-25.0) |
