# Supplementary material for: Dosimetric comparison of hippocampal-sparing technologies in patients with low-grade glioma
Source: Neurooncol Adv. 2024 Aug 6;6(1):vdae131. doi: 10.1093/noajnl/vdae131 (PMC11364934; doi:10.1093/noajnl/vdae131)
Supplement: vdae131_suppl_Supplementary_Appendix_S4 [file vdae131_suppl_supplementary_appendix_s4.docx]

Appendix 4; Dosimetric comparison of contralateral plans (median & IRQ)

|  | VMAT | VMAT_HS | MCO_HS | HyperArc |
| --- | --- | --- | --- | --- |
| Brain -PTV mean dose <57Gy | 19.1 (15.5-22.5) | 19.5 (16.0-23.0) | 19.5 (15.2-22.1) | 18.5 (15.0-21.3) |
| Brain-PTV D10 <24Gy | 36.2 (33.2-40.0) | 37.5 (34.5-40.7) | 37.6 (33.6-42.1) | 35.7 (31.1-38.7) |
| Brainstem mean <52Gy | 28.3 (18.4-34.0) | 27.5 (16.4-33.4) | 26.8 (14.5-32.1) | 26.4 (20.1-34.2) |
| Brainstem D5 <57Gy | 49.6 (46.2-50.7) | 49.5 (45.2-51.0) | 48.4 (43.1-50.2) | 50.2 (44.7-50.7) |
| Chiasm D1 <54Gy | 50.7 (49.5-51.2) | 50.7 (49.2-51.1) | 48.7 (45.8-49.9) | 50.9 (49.9-51.0) |
| Contra LENS D1 <6Gy | 5.5 (5.2-6.0) | 5.8 (5.4-6.3) | 5.3 (4.9-5.7) | 5.1 (4.8-5.5) |
| Lateral LENS D1 <6Gy | 5.6 (4.8-6.0) | 5.7 (5.4-6.4) | 5.3 (4.6-6.3) | 5.4 (5.2-6.0) |
| Contra OPTIC nerve D1 <54Gy | 29.1 (19.0-39.2) | 34.5 (19.4-40.0) | 32.6 (14.0-35.6) | 23.0 (12.1-41.2) |
| Lateral OPTIC nerve D1 <54Gy | 50.0 (31.8-50.5) | 49.6 (32.6-51.0) | 47.2 (27.9-48.6) | 50.2 (31.7-51.1) |
| Contra ORBIT D1 <30Gy | 16.9 (11.7-21.4) | 18.0 (14.3-22.7) | 16.9 (12.4-20.2) | 8.8 (6.8-12.5) |
| Lateral ORBIT D1 <30Gy | 25.4 (15.4-27.0) | 25.6 (17.4-27.8) | 21.2 (14.6-25.6) | 21.0 (15.2-25.4) |
