# Supplementary material for: Dosimetric comparison of hippocampal-sparing technologies in patients with low-grade glioma
Source: Neurooncol Adv. 2024 Aug 6;6(1):vdae131. doi: 10.1093/noajnl/vdae131 (PMC11364934; doi:10.1093/noajnl/vdae131)
Supplement: vdae131_suppl_Supplementary_Appendix_S5 [file vdae131_suppl_supplementary_appendix_s5.docx]

Appendix 5: Spearman correlations (rho with p-values) between PTV size and D40 & Mean dose for contralateral and bilateral hippocampus

|  | Contralateral (n=16) | |  |  | Bilateral (n=9) | |  |  |
| --- | --- | --- | --- | --- | --- | --- | --- | --- |
|  | D40 Contra |  | Mean BiLat |  | D40 Contra |  | Mean BiLat |  |
|  | rho | p | rho | p | rho | p | rho | p |
| VMAT | 0.359 | 0.172 | 0.221 | 0.412 | 0.3 | 0.433 | 0.1 | 0.798 |
| VMAT_HS | 0.724 | 0.002 | 0.485 | 0.057 | 0.233 | 0.546 | 0.117 | 0.765 |
| MCO_HS | 0.638 | 0.008 | 0.462 | 0.072 | 0.233 | 0.546 | 0.183 | 0.637 |
| Hyperarc | 0.679 | 0.004 | 0.606 | 0.013 | 0.367 | 0.332 | 0.367 | 0.332 |
