# Supplementary material for: Dosimetric comparison of hippocampal-sparing technologies in patients with low-grade glioma
Source: Neurooncol Adv. 2024 Aug 6;6(1):vdae131. doi: 10.1093/noajnl/vdae131 (PMC11364934; doi:10.1093/noajnl/vdae131)
Supplement: vdae131_suppl_Supplementary_Appendix_S6 [file vdae131_suppl_supplementary_appendix_s6.docx]

|  |  | D40 (Gy) | | | Mean (Gy) | | |
| --- | --- | --- | --- | --- | --- | --- | --- |
| Location | Plan | location | not location | p-value | location | not location | p-value |
| temporal | VMAT | 25.6 (20.3-29.3) | 22.1 (17.5-23.3) | 0.33 | 25.6 (20.629.3) | 22.4 (19-23.4) | 0.45 |
|  | VMAT_HS | 10.5 (9.0-12.1) | 9.5 (8.3-15.1) | 0.75 | 9.8 (7.6-11.0) | 10.0 (8.6-12.5) | 0.66 |
|  | MCO_HS | 9.2 (7.7-11.4) | 8.2 (6.1-13.1) | 0.83 | 7.8 (6.7-9.7) | 8.6 (6.1-11.9) | 0.83 |
|  | HYPERARC | 10.0 (9.0-10.8) | 7.5 (4.5-13.1) | 0.66 | 8.9 (7.5-10.2) | 7.7 (4.6-11.8) | 0.91 |
| parietal | VMAT | 23.2 (17.8-29.2) | 23.5 (17.8-29.2) | 0.87 | 23.3 (20.629.3) | 21.2 (14.627.8) | 0.53 |
|  | VMAT_HS | 10.0 (8.3-12.6) | 12.0 (11.7-12.1) | 0.34 | 9.8 (8.1-11.3) | 9.0 (7.0-11.0) | 0.63 |
|  | MCO_HS | 8.5 (6.1-10.6) | 11.5 (11.3-11.7) | 0.20 | 7.8 (6.1-10.0) | 9.0 (6.7-11.3) | 0.87 |
|  | HYPERARC | 9.0 (4.7-10.8) | 13.0 (10.1-15.9) | 0.20 | 8.4 (4.8-10.7) | 10.3 (9.4-11.3) | 0.34 |
| frontal | VMAT | 22.1 (17.6-26.3) | 25.6 (21.7-30.6) | 0.29 | 22.4 (17.025.6) | 25.9 (20.830.8) | 0.25 |
|  | VMAT_HS | 10.8 (8.6-13.6) | 10.2 (8.6-11.6) | 0.75 | 10.0 (7.8-11.7) | 9.8 (7.8-10.8) | 0.92 |
|  | MCO_HS | 10.3 (6.6-12.4) | 8.5 (6.8-10.0) | 0.53 | 8.6 (6.4-11.6) | 7.8 (6.7-9.2) | 0.75 |
|  | HYPERARC | 10.3 (4.5-14.5) | 9.0 (6.7-10.4) | 0.67 | 10.1 (4.6-11.6) | 8.3 (6.5-10.1) | 0.60 |

Appendix 6: results of the analysis of the influence of the tumour localisation on the hippocampal mean and D40

Values are medians and IQRs. P-values using Mann Whitney test for differences in medians between location and not

location. i.e. temporal versus not temporal
